# Retraction: CuO and CeO_2_ Nanostructures Green Synthesized Using Olive Leaf Extract Inhibits the Growth of Highly Virulent Multidrug Resistant Bacteria

**DOI:** 10.3389/fphar.2019.00135

**Published:** 2019-02-07

**Authors:** Frontiers Editorial Office

**Affiliations:** ^1^Frontiers Media SA, Lausanne, Switzerland

The journal retracts the September 2018 article cited above. Following concerns identified post-publication, the article was examined by the Chief Editors, confirming image manipulation in Figure 6, as well as re-use from a previous publication and discrepancies in the data sets presented.

The Chief Editors therefore concluded that the article warranted retraction. The authors agree to the retraction.

